# A Case Study of Brass Foundry Workers’ Estimated Lead (Pb) Body Burden from Different Exposure Routes

**DOI:** 10.1093/annweh/wxaa061

**Published:** 2020-06-22

**Authors:** Anneli Julander, Klara Midander, Sandra Garcia-Garcia, Per Vihlborg, Pål Graff

**Affiliations:** 1 Unit of Integrative Toxicology, Institute of Environmental Medicine, Karolinska Institutet, Stockholm, Sweden; 2 Department of Occupational and Environmental Medicine, Faculty of Medicine and Health, Örebro University, Örebro, Sweden; 3 National Institute of Occupational Health, Majorstuen, Oslo, Norway

**Keywords:** biological monitoring, chemical analysis, cutting fluid, dermal exposure measurement, inhalable dust, lead

## Abstract

**Objectives:**

The most pronounced occupational exposure routes for lead (Pb) are inhalation and gastrointestinal uptake mainly through hand-to-mouth behaviour. Skin absorption has been demonstrated for organic Pb compounds, but less is known about inorganic Pb species. Several legislative bodies in Europe are currently proposing lowering biological exposure limit values and air exposure limits due to new evidence on cardiovascular effects at very low blood Pb levels. In light of this, all exposure routes in occupational settings should be revisited to evaluate how to lower the overall exposure to Pb.

**Methods:**

The aim of the study was to investigate the possible exposure routes in workers operating computer numerical control-machines in a brass foundry and specifically to understand if metal cutting fluids (MCFs) used by the workers could lead to skin absorption of Pb. The different bronze alloys at the facility may contain up to 20% Pb. After obtaining written informed consent from the workers (*n* = 7), blood, skin wipes, and personal air samples were collected. In addition, MCFs used on the day of exposure measurements were collected for *in vitro* skin absorption studies using stillborn piglet skin mounted in static Franz diffusion cells (*n* = 48). All samples were analysed for Pb content using inductively coupled plasma mass spectrometry.

**Results:**

Pb air concentration (<0.1–3.4 µg m^−3^) was well below the Swedish occupational exposure limit value. Blood Pb was in the range of <0.72–33 µg dl^−1^, and Pb on skin surfaces, after performing normal work tasks during 2 h, was in the range of 0.2–48 µg cm^−2^. Using the MCFs in diffusion cells showed that skin absorption had occurred at very low doses, and that up to 10% of the Pb content was present in the skin after 24 h exposure. Using these results in the US EPA adult lead model, we could estimate a contribution to blood Pb from the three exposure routes; where hand-to-mouth behaviour yielded the highest contribution (16 µg Pb dl^−1^ blood), followed by skin absorption (3.3–6.3 µg Pb dl^−1^ blood) and inhalation (2.0 µg Pb dl^−1^ blood).

**Conclusions:**

This case study shows that MCF may lead to skin absorption of inorganic Pb and contribute to a systemic dose (quasi-steady state). Furthermore, even though good hand hygienic measures were in place, the workers’ skin exposure to Pb is in all likelihood an important contributor in elevating blood Pb levels. Skin exposure should thus be monitored routinely in workers at facilities handling Pb, to help reducing unnecessary occupational exposure.

## Introduction

Toxicity of lead (Pb) is a well-known problem which was described already by Hippocrates. Today several acute and chronic effects are well documented in the literature. In acute poisoning, both the central and peripheral nervous system are affected, the blood formation is inhibited, kidneys are injured, and severe stomach pain may occur (Mason *et al.*, 2014; Mitra *et al.*, 2017). For long-term exposure, at levels that do not give acute effects, there is a risk of peripheral nervous system effects, cardiovascular effects such as hypertension, and also kidney and immunological effects (Navas-Acien *et al.*, 2007; Gambelunghe *et al.*, 2016). Recent studies have also shown that Pb exposure may be associated with increased cancer risks (Liao *et al.*, 2016; Steenland *et al.*, 2017).

The most common exposure routes for Pb are inhalation and gastrointestinal uptake (Montelius, 2005). Approximately 30–40% of an inhaled dose is absorbed to the bloodstream, whereas gastrointestinal absorption varies depending on the nutritional state (Papanikolaou *et al.*, 2005). It is well known that skin contamination with Pb may lead to elevated blood Pb levels due to hand-to-mouth behaviour (Cherrie *et al.*, 2006; Gorman Ng *et al.*, 2017). The transfer efficiency of a skin dose to saliva has been estimated to be in the range of 12–34% of the original skin dose (Sahmel *et al.*, 2015), indicating that a substantial amount of lead may be available for gastrointestinal uptake.

Regarding skin absorption organic Pb species has been demonstrated to permeate the skin (Franken *et al.*, 2015), whereas it has been hypothesized that inorganic Pb is mainly present in saliva and sweat (Lilley *et al.*, 1988). This may though be challenged by studies in occupational settings where associations for Pb have been found between blood and skin doses (Sun *et al.*, 2002; Sato and Yano, 2006). Also, an *in vitro* study using human skin in diffusion cells, showed that skin absorption of lead oxide (PbO) occurred and that the absorption increased if the skin barrier was damaged (Filon *et al.*, 2006).

Pb exposure occurs in several different work environments, such as lead smelting plants, brass and bronze foundries, glassworks, manufacturing and recycling of batteries, machining of lead mons, and dismantling of electrical waste (Montelius, 2005; Julander *et al.*, 2014; Gorman Ng *et al.*, 2017). Occupational exposure to Pb has traditionally been monitored by air measurement and blood Pb level determination with little emphasis on skin exposure, despite the fact that WHO indicates that skin exposure is one of the major health risks of European workplaces (WHO, 2014), and several studies state that dermal exposure should not be neglected (Behroozy, 2013; Anderson and Meade, 2014; Gorman Ng *et al.*, 2017; Kettelarij *et al.*, 2018a). With regard to Pb exposure of the skin and its contribution on blood Pb levels, new information suggests that the skin’s role for Pb exposure needs to be investigated in more detail (Lilley *et al.*, 1988; Sun *et al.*, 2002; Cherrie *et al.*, 2006; Filon *et al.*, 2006; Gorman Ng *et al.*, 2017). This is also warranted by several legislative bodies responsible for setting occupational exposure limits are suggesting lowering both air exposure and biological exposure limits. In a recommendation from the Scientific Committee on OELs (SCOEL) already in 2002, it was suggested that a biological exposure limit for Pb of 30 µg dl^−1^ blood should be implemented, although also adding that this level ‘is not seen as being entirely protective of the offspring of working women’ (Scientific Committee on Occupational Exposure Limits, 2002). A proposal from the European Chemicals Agency (ECHA), regarding lowering the biological exposure limit to 15 µg dl^−1^ is currently out on public consultation (European Chemicals Agency, 2019). The proposal clearly states that the limit would not be protective for women in fertile age. In the newest provision for medical surveillance in Sweden the current level for Pb exposed workers is set to 30 µg dl^−1^ for all employees except women under 50 years of age, which may not be exposed to Pb if blood lead levels are above 10 µg dl^−1^ (Swedish Work Environment Authority, 2019).

An opportunity to study the effect of skin exposure to Pb with regard to blood levels presented when a brass foundry in the region of Örebro, Sweden turned to the clinic of Occupational and Environmental Medicine, at the Örebro University Hospital for assistance with mapping workers Pb exposure due to elevated blood Pb levels (range: 21–41 µg dl^−1^). Despite preventive measures such as improvement of local exhaust ventilation on the computer numerical control (CNC)-machines and information on the importance of hand hygiene on regular basis, blood Pb levels had not significantly reduced. In conjunction with this study, metal cutting fluids (MCFs) used at the brass foundry were collected to study possible skin permeation of Pb *in vitro* using Franz diffusion cells.

## Methods

Participation in the project was voluntarily and all participants signed a written consent before taking part in the study. The project was approved by the regional ethical board in Uppsala (permit no. 2014/513).

The study was conducted at a brass foundry that casts and machines plain bearings in different bonze alloys, some of which can contain up to 20% Pb. Seven male CNC-operators were recruited to the study. The mean age was 39 years (range 22–57 years). These workers did not work in the foundry areas of the production facility, and hence did not have any air exposure to Pb as shown by the regular air monitoring performed by the company. During the manufacturing process the workers handle the materials with their hands when loading/unloading the CNC-machines and thus are exposed to the MCF.

During work, all participants used polycotton overalls and some used gloves (rubber). No other personal protective equipment was used during the ordinary work tasks. All workers in this foundry undergo regular surveillance for levels of Pb in blood. If measurements exceeded 41 µg dl^−1^ workers were suspended from Pb exposed work until levels upon retesting is below 37 µg dl^−1^. Thereafter they could resume their ordinary work tasks, in accordance with Swedish legislation (Swedish Work Environment Authority, 2005).

To evaluate the Pb exposure, collection of blood samples, skin wipe samples, and personal air monitoring samples was performed on the same day. Furthermore, samples of the MCFs (currently or previously used) were also collected for quantification of Pb content and to be used in an *in vitro* study of skin absorption of Pb.

### Blood Pb quantification

At the end of shift, approximately 7 ml of venous blood was collected in a VACUETTE® trace element tube with sodium heparin (Greiner Bio-One, Kremsmünster, Austria) from each participant. Before analysis, the blood samples were homogenized on a rotating mixer and equal aliquots (0.5 ml) of blood and a solution of 25% ammonium/1% ethylenediaminetetraacetic acid was mixed and further diluted with MilliQ-water (Selden *et al.*, 2008). Internal standardization was used for quantitative evaluation. The samples were analysed using an Inductive Coupled Plasma Mass Spectrometry (ICP/MS, iCAP™ Q: Thermo Fisher Scientific, Waltham, MA, USA). The limit of detection (LOD) was set to 0.72 µg dl^−1^ (3× standard deviation in blank).

### Air Pb quantification

Personal exposure measurements of inhalable particulate matter (PM) were conducted for 8 h with filters in the breathing zone according to the Methods for the Determination of Hazardous Substances (MDHS 14/3) (HSE, 2000). Sampling of inhalable PM used a 25 mm filter with 3 µm pore size mounted in an IOM sampler, flow 2.0 l min^−1^. Before and after sampling, the filter mounted in the cassette is allowed to stabilize for 3 days in a climate-controlled weighting room (20 ± 1°C and relative humidity 40 ± 2%). After this time period, weighting was done with a Mettler Toledo MX5 microbalance (Mettler Toledo, Greifensee, Switzerland). The limit of quantification for the gravimetric analysis of inhalable PM was 0.5 mg.

Chemical analysis of Pb content in the PM collected on the filter was performed in accordance with the NIOSH method 7300 (NIOSH, 2003). In short, the PM and filter were dissolved in nitric acid, diluted with MilliQ-water, followed by analysis with an ICP-MS for metal determination. The LOD for the measurement of Pb was 0.3 µg.

### Skin surface Pb quantification

To quantify skin exposure to Pb the acid wipe sampling method was used (Lidén *et al.*, 2006). In short, the method uses 0.5 ml of 1% HNO_3_ (not harmful to skin) to wipe skin areas. The procedure is performed with 3 wipes per area. The workers washed their hands with soap and water, thereafter, sampling areas (index finger, palm, and wrist on both hands and non-dominant little finger) were wiped with 1% HNO_3_ and rinsed with ultrapure-water (Milli-Q Gradient; Merck Millipore, Darmstadt, Germany) to remove all metals present on the skin surface. The little finger on the non-dominant hand was masked to act as a clean reference surface.

The workers then performed their normal work tasks for 2 h without washing their hands during this period. Thereafter, areas of 3 × 3 cm were marked on the palms and wrists and areas of 1 × 2 cm were marked on the index fingers (Table 1). Each marked surface was wiped with three pieces of cellulose paper moistened with 0.5 ml of 1% HNO_3_ each (1.5 ml in total). All three acid wipes from the surface were placed in an acid-washed plastic tube (50 ml), which was filled with 23.5 ml of 1% HNO_3_. The flasks were shaken for 30 min, and the liquid was then transferred to a different tube (50 ml). Three unused wipes were treated in the same manner to serve as a blank sample. The extracts were analysed for Pb by ICP/MS.

**Table 1.** Individual values of end-of-shift blood Pb (µg dl^−1^) and Pb skin doses (µg cm^−2^) accumulated during 2 h for palm, wrist, and index finger on the left and right hand, respectively. The data on skin doses are corrected for possible Pb content on the reference surface. Data are approximated to 1 decimal.

Worker	Blood Pb (µg dl^−1^)	Left hand			Right hand		
		Palm (µg cm^−2^)	Wrist (µg cm^−2^)	Index (µg cm^−2^)	Palm (µg cm^−2^)	Wrist (µg cm^−2^)	Index (µg cm^−2^)
1	14.9	3.8	0.8	12	4.5	1.4	15.3
2	6.0	0.6	0.2	0.7	0.8	0.2	0.9
3	29.0	4.1	1.1	24.6	8.0	0.8	32.4
4	33.2	9.1	3.4	36.4	13.8	3.0	48.4
5	7.4	4.2	0.4	7.1	2.3	0.6	9.5
6	4.9	1.4	2.4	3.7	0.9	3.1	6.5
7	<0.7^a^	0.2	0.1	1.0	0.52	0.6	4.6

^*a*^Below LOD; for statistical analysis the value was divided by square root of 2.

### MCF Pb quantification

Samples of the MCFs used in the CNC-machines, both from the day of sampling and from 1 month before the day of sampling, were sent to ALS Scandinavia AB for the determination of Pb. The samples were analysed by ICP/SFMS (Sector Field ICP/MS) according to EPA-Method 200.8 and SS EN ISO 17294-1.

### *In vitro* skin absorption of Pb

Experimental skin absorption of Pb from MCFs was studied in the laboratory following a standardized method (OECD guideline no 428, 2004), using skin from stillborn piglets. One exception from OECD guideline was made: no sampling from the receptor was done during runtime of the experiment to avoid risk of dilution of the Pb concentration to below LOD in chemical analysis. No ethical permission is needed since the piglets were not bred for research purposes and died of natural causes.

The experimental set-up allowed five parallel exposures in static diffusion cells (Franz cells, Permegear, Bethlehem, PA, USA) with an orifice of 9 mm in diameter, corresponding to an exposure area of 0.64 cm^2^. The receptor compartment volume was 5.0–5.4 ml and the donor compartment volume 1 ml. The diffusion cells were placed on a magnetic stirring plate (HP 6 Variomag, H+P labortechnik, Munich, Germany) and connected to a thermostatic water bath (21 AT, Heto, Alleod, Denmark) regulating the temperature at 32°C. Five ml phosphate buffered saline (PBS) solution (Filon *et al.*, 2006) and a teflon magnetic stirring bar were added to receptor compartments prior to exposure.

Pieces sized 3 × 3 cm were cut from frozen full skin with a sterile scalpel (Kiato, Sylak AB, Askim, Sweden). Transepidermal water loss (TEWL, Dermalab, Cortex Technology) was measured for 50 s to confirm acceptable barrier integrity (<10 g m^–2^ h^–1^) after thawing at room temperature. Skin thickness was measured using an Oditest tool (H.C. Kroeplin GmbH, Schlüchtern, Germany). Thereafter, the skin samples were placed in 15 ml PBS to rehydrate for 15 min prior to the experiment.

Skin was carefully rolled onto the receptor chamber using tweezers, and the donor compartment was mounted on top using metal clamps, making sure no bubbles were trapped underneath skin in the receptor compartment.

The study was designed to test four MCFs and one blank (MilliQ-water) on skin from four different piglets in three experiments at 2, 4, and 24 h. Prior to the start of the experiment the MCF was shaken for 30 s before 800 µl (infinite dose) of the MCF was added to the respective diffusion cell. In one diffusion cell 800 µl of MilliQ-water was added as a blank to each experiment. After adding the donor, the diffusion cell was sealed with parafilm in order to avoid evaporation during the experiment. At the same time as loading of donors, three additional samples of each MCF were taken from the container and put into three polypropylene-plastic tubes (12 ml, Sarstedt, Nümbrecht, Germany) to establish start concentration of Pb by metal analysis.

At the end of exposure of each time point, the remaining donor solution (MCF) was collected using Pasteur pipettes and transferred to test tubes. The donor was rinsed with 3 × 1 ml of MilliQ-water and collected into test tubes. The entire receptor media, 5 ml, was collected in the same way, followed by rinsing with 5 ml of 1% HNO_3_ and added to the test tube. The exposed skin was detached from the diffusion cell and rinsed with MilliQ-water. The skin was placed in a Petri dish to record the different measures of barrier integrity (TEWL and resistance). A skin punch sample, 8 mm in diameter, was collected from the exposed skin area using a skin punch (Kai Europe GmbH, Solingen, Germany). The skin sample was then placed in a micro test tube (2.5 ml, Sarstedt, Nümbrecht, Germany) and 1 ml 67% HNO_3_ (Normatom, VWR BdH Chemicals, Leuven, Belgium) was added to dissolve the skin while placed in a fume hood (for at least 72 h) awaiting further sample preparation and metal analysis.

Throughout the experimental study, MilliQ-water was used to prepare PBS and diluted HNO_3_ and to rinse exposed skin and clean the diffusion cells. All materials used in sample preparations (test tubes, etc.) were acid washed (soaked at 40°C in 10% HNO_3_ and rinsed four times in deionized water, 16.8 MΩ cm^−1^). Material and instruments in contact with skin samples were surface cleaned by ethanol (96% vol, GPR RECTAPUR, VWR International, Fontenay-sous-Bois, France).

Chemical analysis of Pb content in donors, donor rinses, skin punch samples, receptors, receptor rinses, and MCFs was performed using ICP-MS (iCAP Q Thermo Fisher Scientific, Qtegra version 2.10). The ICP-MS was run in kinetic energy discrimination mode using argon as vector gas and helium as a collision gas. As an internal standard indium (In, 1000 µg ml^−1^ in 2% HNO_3_, Spectrascan, Teknolab, Ski, Norway) was added at 5 ppb to all samples. A 7-point calibration curve was used. The LOD (3× the standard deviation of the blank samples) was <0.06 ppb for all runs.

### Data analysis

All statistical analyses and graphs were prepared using GraphPad Prism, version 8.3.0. Since the study only has limited data, all analyses have been made using non-parametric tests. To evaluate the association between blood Pb and Pb on skin Spearman’s Rho correlation was performed. Kruskal–Wallis test with Dunn’s test for multiple comparisons was used to evaluate differences between the different skin surfaces that were sampled for Pb. Furthermore, we used Kruskal–Wallis signed rank test, to evaluate differences between the levels of Pb absorbed in skin from the MCF in the *in vitro* study. A *P*-value of 0.05 was considered significant.

To estimate the possible contribution to blood Pb levels from skin absorption, we used the data from the *in vitro* study and the mean value of Pb skin doses collected from the workers hands, together with the US EPA adult lead model (US EPA, 2003). As input values we use 220 workdays per year, a total hand surface for two hands of 1070 cm^2^ (US EPA, 2011) and a biokinetic slope factor (quasi-steady state blood level) of 0.4 µg Pb dl^−1^ per 1 µg Pb absorbed day^−1^ (US EPA, 2003). For calculations we used the average skin dose of all sampled skin surfaces after 2 h work (6.5 µg cm^−2^) from the seven participants as input for skin dose (µg day^−1^) in the model, by multiplying the average skin dose with total hand surface. This adaption was made since we did not have accumulated 8-h skin dose form the workers. Furthermore, we also used the same method (US EPA, 2003), together with literature data from Sahmel *et al.* (2015), Cherrie *et al.* (2006), US EPA (2011), and Gorman Ng *et al.* (2016) to estimate the contribution to blood Pb from hand-to-mouth activity. To get an estimate of blood Pb levels, we assumed that 13.4 cm^2^ skin surface on hands is in contact with the peri-oral area, that 24% is transferred from hand-to-mouth and 20% is subsequently absorbed in the gastrointestinal tract. To estimate the contribution from inhalation we used the average Pb concentration in inhalable PM measured in this study together with literature data on volume of inhaled air during 8 h (10 m^3^) and lung absorption of inhalable fraction (70%) (Cherrie *et al.*, 2006).

## Results

We present results showing that Pb was present in blood, air, and on skin surfaces among the workers participating in this study. Furthermore, we show that Pb from MCFs permeates piglet skin and that substantial amounts of Pb were present in the skin after exposure.

### Occupational study

The Pb content in the MCFs collected from the CNC-machines at the day of exposure measurements varied between 29 and 132 mg kg^−1^. Samples of unused MCFs had a low Pb content (<4 mg kg^−1^). Samples of older MCFs, not used on the day of sampling, had even higher amounts of lead (226–453 mg kg^−1^), indicating that the exposure may be even higher than measured in this study.

Air concentrations of inhalable PM were all below the detection limit (<0.5 mg) with the gravimetric method. The inhalable PM had a Pb content ranging from <0.1 to 3.4 µg m^−3^ (mean 1.2 ± 1.1 µg m^−3^), all well below the Swedish OEL (0.1 mg m^−3^).

**Figure 1.**
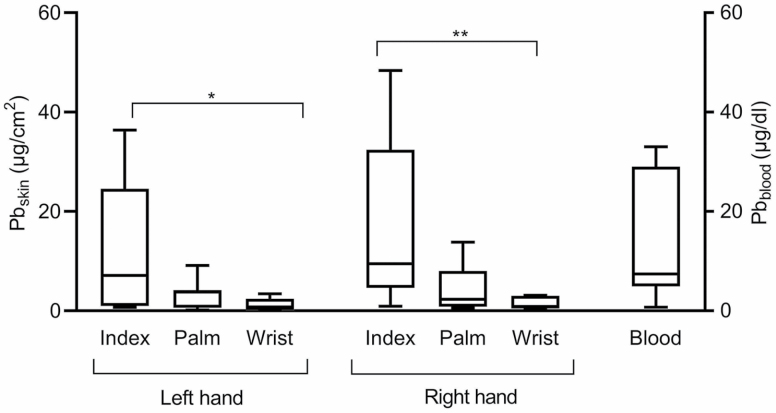
Box and whisker graph displaying median value and 5–95th percentile of skin dose on individual fingers (µg cm^−2^) of the left- and the right hand presented on the right *y*-axis and blood Pb (µg dl^−1^) on left *y*-axis in seven participants. Note the difference in the units on the *y*-axis. There is significantly more Pb present on the index fingers than on the wrist of both hands (Kruskal–Wallis test with Dunn’s multiple comparison test). *P < 0.05; **P < 0.01

Blood Pb levels among the workers ranged from <0.72 to 33 µg dl^−1^ (Table 1), and Pb on skin was found on all the sampled surfaces of the workers’ hands (0.16–48 µg cm^−2^). The Pb content on the skin varied greatly between surfaces on the hands and between workers (Table 1). Furthermore, evaluating the surfaces of the two hands, the index fingers of both hands were the most contaminated ones (Fig. 1) and compared with the wrists there was significantly more Pb on the fingers.

**Figure 2.**
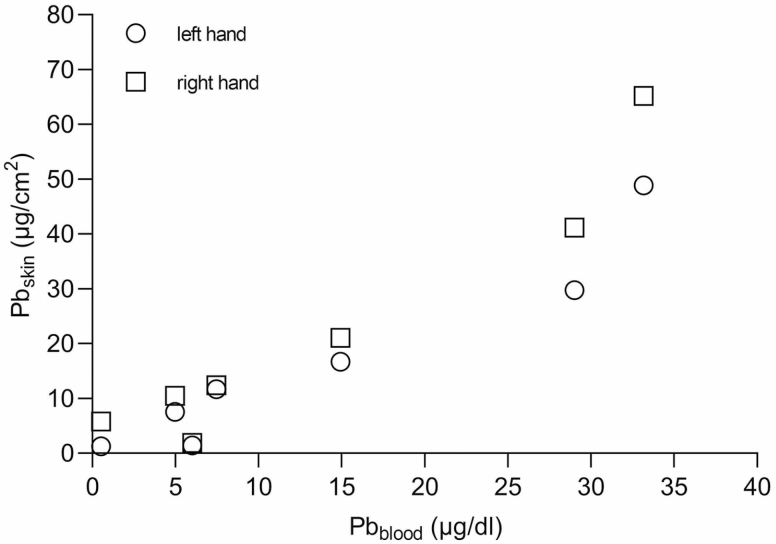
The association between Pb in blood (µg dl^−1^) versus sum of Pb on all sampled surfaces on skin (µg cm^−2^) for each hand of the seven participants. Right hand data are indicated by squares and left hand data by circles. Blood levels correlate to skin dose of Pb (Spearman’s Rho): left hand *r* = 0.964; *P* = 0.0028 and right hand *r* = 0.892; *P* = 0.0123.

Elevated blood Pb levels were found in two of the study participants (29 and 33 µg dl^−1^) compared with the other study subjects (<0.72–14 µg dl^−1^) (Table 1). These two workers also had higher amounts of Pb on their hands than the rest of the workers, possibly as a result of not wearing gloves. A Spearman’s Rho correlation analysis was made to evaluate the association between blood Pb levels and skin Pb doses (sum of sampled areas). The analysis showed a high positive correlation between blood Pb levels and skin dose of Pb; left hand *r* = 0.964; *P* = 0.0028 and right hand *r* = 0.892; *P* = 0.0123 (Fig. 2).

### *In vitro* diffusion cell study

**Figure 3.**
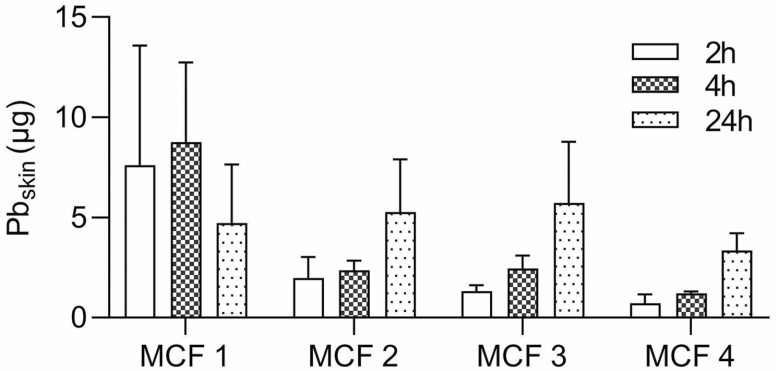
Accumulation of Pb in skin (µg) at the three different time points. For all MCFs, except MCF 1, there seem to be a time-dependent increase of Pb accumulated in skin, indicating the build-up of a reservoir in the skin.

The results of the experimental skin absorption tests of Pb from the MCFs collected from the CNC-machines are shown in Table 2. The amount in the four different MCFs ranged between 48 and 290 µg cm^−2^ Pb at the start of the experiment. From Table 2 it is evident that more Pb was present in skin than in the receptor compartment, although most of the Pb was still found in the donor compartment. Fig. 3 shows the amount of Pb (µg) that accumulated in the skin after 2, 4, and 24 h. There is a trend for three of the MCFs to have higher amounts of Pb in skin after 24 h, although this is not statistically significant (Kruskal–Wallis signed rank test), indicating a build-up of a Pb reservoir in the skin.

**Table 2.** Average amounts of Pb in µg cm^−2^ (%) quantified in the MCF donor matrix before start of experiment, and in donor, exposed skin and PBS receptor media after 2, 4, and 24 h of exposure.

Sample	Time of exposure	*n*	MCF 1	MCF 2	MCF 3	MCF 4
			µg cm^−2^ (%)	µg cm^−2^ (%)	µg cm^−2^ (%)	µg cm^−2^ (%)
MCF matrix	0 h	12	290 (100)	79.2 (100)	97.8 (100)	48.4 (100)
Donor	2 h	4	172 (59.3)	60.0 (75.8)	62.2 (63.5)	33.8 (69.9)
	4 h	4	109.4 (37.7)	36.2 (45.6)	69.0 (70.5)	37.2 (76.5)
	24 h	4	146.4 (50.5)	53.2 (67.2)	63.6 (64.9)	29.0 (59.8)
Skin	2 h	4	12.0 (4.13)	3.10 (3.91)	2.07 (2.11)	1.13 (2.33)
	4 h	4	13.8 (4.76)	3.73 (4.71)	3.86 (3.95)	1.91 (3.94)
	24 h	4	7.41 (2.55)	8.28 (10.4)	9.00 (9.20)	5.27 (10.9)
Receptor	2 h	4	0.00248 (0.001)	0.00197 (0.002)	0.00278 (0.003)	0.000826 (0.002)
	4 h	4	0.00244 (0.001)	0.00218 (0.003)	0.00276 (0.003)	0.00104 (0.002)
	24 h	4	0.00324 (0.001)	0.00238 (0.003)	0.00438 (0.004)	0.00306 (0.002)
			MCF 1	MCF 2	MCF 3	MCF 4
Average diffusion rate^a^ (ng cm^−2^ h^−1^)	0–2 h	4	0.97	0.77	1.09	0.32
	0–4 h	4	0.47	0.43	0.54	0.20
	0–24 h	4	0.11	0.077	0.14	0.10

^*a*^Average diffusion rate calculated as: mass Pb in receptor (µg)/area exposed skin (cm^2^) × time (h).

### Contribution to blood Pb from different exposure routes

The steps in our calculations to estimate the contribution to blood Pb levels from inhalation, skin absorption and transfer from hand-to-mouth are presented in Table 3. The results clearly show that hand-to-mouth behaviour gives the highest contribution to blood Pb (16.2 µg dl^−1^), followed by skin absorption (3.44–6.33 µg dl^−1^) and inhalation (2.02 µg dl^−1^).

**Table 3.** Calculated contribution to blood Pb concentration in three cases. Case 1 is skin absorption using average skin dose of workers (during 2 h) and skin absorption (2 and 24 h values, respectively) from the *in vitro* study of MCF. Case 2 is hand-to-mouth transfer using average skin dose of workers (during 2 h). Case 3 is inhalation exposure using mean value of Pb air concentration from this study.

	Case 1	Case 2	Case 3	References
	Skin absorption (2/24 h)	Hand-to-mouth inadvertent ingestion	Inhalation	
Average air Pb concentration (µg m^−3^)			1.2	This study
Inhaled air volume during 8 h (m^3^)			10	Cherrie *et al.* (2006)
Lung absorption (%)			70	Cherrie *et al.* (2006)
Average skin dose (µg cm^−2^) during 2 h	6.5	6.5		This study
Skin surface (cm^2^)	1070	13.4		US EPA (2011)
Skin absorption (%)	0.00197/0.00374			This study
Contact frequency (events h^−1^)		2		This study and Cherrie *et al.* (2006), Gorman Ng *et al.* (2016)
Transfer efficiency into mouth		24%		Sahmel *et al.* (2015)
Gastrointestinal absorption		20%		Papanikolaou *et al.* (2005)
Uptake dose (µg day^−1^)	13.8/26.3	67.5	8.4	Calculated this study
BKSF (µg dl^−1^ per µg day^−1^)^a^	0.4	0.4	0.4	US EPA (2011)
Workdays	220	220	220	5–6 weeks’ vacation year^−1b^
Blood lead (µg dl^−1^)^c^	3.34/6.33	16.2	2.02	Calculated this study

^*a*^BKSF = ‘Biokinetic slope factor relation (quasi-steady state) increase in a typical adult blood lead concentration to average daily lead intake (µg/dL blood increase per µg/day lead uptake).’ (US EPA, 2003).

^*b*^The Confederation of Swedish Enterprise (FOLA, 2019).

^*c*^Calculated value based on the US EPA adult lead model.

## Discussion

In this study, we present novel data from workers’ exposure to Pb originating from MCF. By measuring Pb in blood, skin wipes, and air samples in conjunction with performing *in vitro* studies of skin absorption of Pb from the MCF, we have been able to estimate a body burden from three exposure routes: inhalation, skin absorption, and inadvertent ingestion.

Occupational skin exposure to Pb and its association to blood, urine, or sweat levels has been studied previously (Lilley *et al.*, 1988; Dykeman *et al.*, 2002; Sun *et al.*, 2002; Sato and Yano, 2006). Our data on skin exposure of Pb and correlations to blood Pb levels confirm the association found in battery workers by Sun *et al.* (2002). In the latter study, skin doses of Pb were in the range of 1.72–7.21 µg cm^−2^ collected only on the dorsal side of the hand, which is comparable to the levels measured in our study (0.01–24 µg cm^−2^). The blood Pb values found by Sun *et al.* (10–54 µg dl^−1^) are comparable, but slightly higher, to those found in our study (4.9–33 µg dl^−1^). This could perhaps be explained by the lack of sampling more contaminated areas of the hands, such as palm and fingertips in the Sun *et al*. study. In addition to blood levels and skin doses of Pb, the air concentration of Pb (inhalable PM) in our study is low (1.2 µg m^−3^, corresponding to 1% of Swedish OEL). Consequently, Pb skin doses were of interest given the issue of elevated blood Pb levels in the workers.

Organic forms of Pb have been shown to penetrate the skin (Franken *et al.*, 2015), while skin absorption of inorganic Pb is generally considered negligible (WHO, 1995; Cherrie *et al.*, 2006). For example, the flux of tetrabutyl lead (C_16_H_36_Pb) is 20 µg cm^−2^ h^−1^ at 24 h, whereas PbO is less than 30 ng cm^−2^ h^−1^ as reviewed by Franken *et al.* (2015). In our study, the average diffusion rate was calculated to 0.78 ng cm^−2^ h^−1^ for the 2-h experiment, 0.41 ng cm^−2^ h^−1^ for the 4-h experiment, and finally 0.11 ng cm^−2^ h^−1^ for the 24-h experiment. Due to the experimental procedure we do not calculate a flux, which is the change in mass for a specific time interval, hence the numbers are not directly comparable to the limited data in the literature. Applying our calculation method to the data in experiment 1 (24 h absorption with PbO) presented in Filon *et al.* (2006) the diffusion rate will be 0.12 ng cm^−2^ h^−1^, which is almost exactly the same number as in our study for 24 h. These data indicate that the Pb in our MCF do permeate the skin, but at very slow rates compared with organic Pb.

Since the flux/diffusion rate through skin for inorganic Pb is low, it has been suggested that skin contamination by Pb is mainly important from the perspective of inadvertent ingestion, i.e. hand-to-mouth behaviour. A study of transfer efficiency of a Pb dose from finger to saliva showed that a substantial amount of Pb (12–34% of a skin dose) may be ingested this way (Sahmel *et al.*, 2015). To prevent gastrointestinal uptake of lead, which can be as high as 30–70% depending on nutritional status (Papanikolaou *et al.*, 2005; Cherrie *et al.*, 2006), it is recommended that workers clean their hands often and always before eating.

However, as shown in this study a substantial amount of Pb is present in the skin already after 2 h exposure and is increasing to 24 h. This build-up of lead in the skin indicates a possible reservoir effect which must be considered. There is limited data on the fate of reservoirs of metals in skin, but the study by Filon *et al.* (2006), show that rapid decontamination of skin with soap (30 min after start of exposure), does not lower the level of Pb in the receptor compartment at 24 h. This indicates that a reservoir in skin is quickly built up and continues to contribute to Pb permeation through skin during at least 24 h.

In an effort to better understand the different exposure pathways, we estimate the body burden of Pb using the adult lead model presented by US EPA (2003). As input data we used the *in vitro* absorption data (at 2 and 24 h) and data on skin and inhalation exposures measured in this study. When performing calculations some assumptions will have to be made. In our study, we consider it a strength that we have measured skin doses as well as inhalation concentrations of Pb from the workers. Another strength is that we have used the MCFs used by the workers to set the absorption value for skin permeation of Pb. One weakness is that we did not study the hand-to-mouth behaviour in the workers, so we had to set a value based on literature and recommended hygiene practice at the foundry. However, we do know that the workers always washed their hands before eating and breaks. In a review by Cherrie *et al.* (2006) the authors provide a thorough inventory of the literature on hand-to-mouth frequency, that pinpoint the importance of inadvertent ingestion of hazardous substances and motivate the estimation of 5 contacts h^−1^ for calculations of ingested dose of Pb, with the comment that it is probably a rather high frequency (Cherrie *et al.*, 2006). In another study by Gorman Ng *et al.* (2016) the authors showed that when workers wear gloves, the contact frequency may be as low as 1.2 contacts h^−1^. Taking this into consideration we decided to use 2 contacts h^−1^ since the workers often wear gloves are well-trained in cleaning their hands during a workday and, also well aware that they should not touch the peri-oral area with their hands. The estimation of quasi-steady state contribution to body burden of Pb, presented in Table 3, shows that hand-to-mouth behaviour gives the highest contribution to blood Pb (16.2 µg dl^−1^), followed by skin absorption (3.44–6.33 µg dl^−1^) and inhalation (2.02 µg dl^−1^). Worth noting is that the skin absorption values were calculated using an average skin dose from five workers wearing gloves and two workers not wearing gloves. These two workers inevitably pull the mean value of skin dose up, however even without the addition of their skin doses, the skin absorption will contribute with 1.54–2.91 µg dl^−1^. This indicates that skin exposure must be reduced in Pb exposed workers to be able to lower the blood Pb levels further, especially since the body of evidence from different legislative agencies shows that there is no agreement on where, if at all, a threshold could be found for Pb-induced health effects in the working population (Scientific Committee on Occupational Exposure Limits, 2002; European Chemicals Agency, 2019; Swedish Work Environment Authority, 2019).

How to reduce skin exposure needs to be carefully evaluated. The simplest way of reducing skin contamination is to increase the frequency of hand washing or to use gloves. In our study two workers did not use gloves, resulting in higher Pb levels on the skin. In addition, some workers may not be able to wear gloves during some operations, such as manual grinding, polishing, and turning as it may be a risk of injury if the glove gets stuck in the machine (Julander *et al.*, 2010). But more often incorrect use of protective gloves occurs, and the wrong type of gloves is used so that the skin is not protected. A recently published study show that workers’ glove use did not affect the amount on cobalt deposited on skin in a hard metal facility due to the factors mentioned above (Kettelarij *et al.*, 2018b). Furthermore, the efficiency of washing off metals from the skin has been investigated in some studies, showing that cobalt, chromium, iron, nickel, and lead, remain on the skin surface even after thorough cleaning (Linnainmaa and Kiilunen, 1997; Sato and Yano, 2006; Liden *et al.*, 2008; Lewinski *et al.*, 2017). In addition, it is well known that repeated and intensive hand washing can damage the skin’s barrier function through dehydration, friction and the influence of chemicals in the soaps used (Hamnerius *et al.*, 2018). Surfactants such as sodium lauryl sulphate (SLS), various scrubbing additives and solvents are often included in soaps and creams for the cleaning of heavily contaminated skin which may be problematic. An *in vitro* study of skin absorption of PbO showed that the uptake of Pb increased eight times if the skin was washed with a soap containing SLS (Filon *et al.*, 2006). In this study, the authors also used the US EPA adult lead model to quantify the addition to body burden of Pb by skin absorption. The result was in the same range as seen in our study, furthermore Filon *et al.* (2006) also showed an increase in body burden if the skin barrier was damaged, which is often the case in industry workers. In many cases with occupational exposure to Pb, the workers are trained to continuously wash their hands as a way of reducing inadvertent oral ingestion. However, this behaviour risks damaging the skin barrier depending on the cleaning method, hence leading to a possible increased skin absorption. In this context, the benefit of increased hand washing should be revisited.

To advise on how to best protect the skin from metal exposure is often more complex than just to recommend increased hand washing or increased glove use. In many instances a combination of methods is required, including the selection of correct gloves, education of personnel on how to use the gloves, in connection to having soaps and moisturizing creams available. Also, it is important to evaluate the effectiveness of the protective measures intended to reduce skin contamination prior to give further recommendations. In the case of protecting the workers from skin absorption of Pb, more research is needed on understanding factors leading to skin permeation.

## Conclusion

This case study shows that skin exposure while working with Pb-containing MCFs may lead to skin contamination and absorption of inorganic Pb contributing to a systemic dose. Furthermore, even though good hand hygienic measures were in place, the workers’ skin doses of Pb were found to play an important role for their elevated blood Pb levels. Skin exposure should thus be monitored routinely in workers at facilities handling Pb, to help reduce unnecessary occupational exposure.
